# Excretion of SARS-CoV-2 through faecal specimens

**DOI:** 10.1080/22221751.2020.1844551

**Published:** 2020-11-25

**Authors:** Yong Zhang, Cao Chen, Yang Song, Shuangli Zhu, Dongyan Wang, Hui Zhang, Guangyue Han, Yuwei Weng, Jun Xu, Jianan Xu, Pengbo Yu, Weijia Jiang, Xianda Yang, Zhongkai Lang, Dongmei Yan, Yanhai Wang, Jingdong Song, George Fu Gao, Guizhen Wu, Wenbo Xu

**Affiliations:** a NHC key laboratory for Medical Virology, NHC key laboratory for biosafety. National Institute for Viral Disease Control and Prevention, Chinese Center for Disease Control and Prevention, Beijing, People’s Republic of China.; b Gansu Provincial Center for Disease Control and Prevention, Lanzhou City, People’s Republic of China; c Hebei Provincial Center for Disease Control and Prevention, Shijiazhuang City, People’s Republic of China; d Fujian Provincial Center for Disease Control and Prevention, Fuzhou City, People’s Republic of China; e Heilongjiang Provincial Center for Disease Control and Prevention, Harbin City, People’s Republic of China; f Sichuan Provincial Center for Disease Control and Prevention, Chengdu City, People’s Republic of China; g ShaanxiProvincial Center for Disease Control and Prevention, Xi'an City, People’s Republic of China.; h Guizhou Provincial Center for Disease Control and Prevention, Guiyang City, People’s Republic of China; i Jilin Provincial Center for Disease Control and Prevention, Changchun City, People’s Republic of China; j Chongqing Wanzhou Center for Disease Control and Prevention, Chongqing City, People’s Republic of China; k Chinese Center for Disease Control and Prevention, Beijing, People’s Republic of China

**Keywords:** COVID-19, faecal specimens, faecal-oral transmission, clinical typing, public health

## Abstract

Coronavirus disease 2019 (COVID-19) has become a pandemic with increasing numbers of cases worldwide. SARS-CoV-2, the causative virus of COVID-19, is mainly transmitted through respiratory droplets or through direct and indirect contact with an infected person. The possibility of potential faecal-oral transmission was investigated in this study. We collected 258 faecal specimens from nine provinces in China and detected the nucleic acid of SARS-CoV-2 using real-time RT–PCR. Vero cells were used to isolate the virus from SARS-CoV-2 nucleic acid positive samples, after which sequencing of Spike gene in eight samples was performed. In all, 93 of 258 (36%) stool samples were positive for SARS-CoV-2 RNA. The positive rates of critical, severe, moderate, and mild patients were 54.4%, 56.1%, 30.8%, and 33.3%, respectively. The content of nucleic acid increased within 2 weeks after the onset of the disease. From the perspective of clinical typing, the nucleic acid can be detected in the faeces of critical patients within two weeks and until four to five weeks in the faeces of severe and mild patients. SARS-CoV-2 was isolated from stool specimens of two severe patients. Four non-synonymous mutations in Spike gene were newly detected in three stool samples. A small number of patients had strong faecal detoxification ability. The live virus in faeces could be an important source of contamination, which may lead to infection and further spread in areas with poor sanitary conditions. The findings of this study have public health significance and they should be considered when formulating disease control strategies.

## Introduction

Known in China as novel coronavirus pneumonia (NCP), the main clinical features of coronavirus disease 2019 (COVID-19) are fever, dry cough, and pneumonia. As of 4 May 2020, COVID-19 had affected more than 84,338 people across China with 4642 deaths, as reported by the National Health Commission of China.

In order to track down the infected pathogens, several public health expert groups gathered to investigate and determine the source of virus transmission. Initially, it was considered that a spill-over took place and the virus was disseminated into the local pool by animal-to-human transmission; however, later investigations confirmed human-to-human transmission of the virus, with people having no history of travelling to Wuhan, the epicentre of COVID-19 outbreak, getting infected with the virus [1].

In the early stage of the disease, the virus load in the respiratory tract samples is relatively high [1]. Initial studies have shown that the disease is mainly transmitted through close contact with infected persons, such as through living together, gatherings, transportation, and public places, indicating that the main transmission of the epidemic is through respiratory droplets and through direct and indirect contact with infected individuals [2,3].

The studies on 2003 SARS outbreak indicated that individuals infected with SARS do not transmit the virus during the incubation period. However, it has been reported that SARS-CoV-2 could be transmitted during the incubation period [4]. This peculiar behaviour of the virus shows that, through constant mutation, SARS-CoV-2 has attained sufficient virulence to be more compatible with the host. Moreover, previous studies of the 2003 SARS outbreak revealed that, despite severe signs and symptoms of the infection, the transmission route of the virus was clear. Thus, it was easier to avert the transmission of the virus [5]. Conversely, SARS-CoV-2 has less severe sign and symptoms, but its mode of transmission is relatively more complicated [6,7].

COVID-19 is a highly infectious disease, and it has spread worldwide. Respiratory symptoms are most common in COVID-19 patients, but these are not the only symptoms associated with SARS-CoV-2 infection. Some COVID-19 patients exhibit less typical symptoms, including nausea and diarrhoea. Several studies have shown that nearly one third of COVID-19 patients have gastrointestinal symptoms, including loss of appetite, nausea, vomiting, or diarrhoea [8–10]. There exist some suspicions that stool shedding or the oral-faecal route might play an important role in the silent transmission of SARS-CoV-2. Therefore, in this study, we investigated stool shedding of SARS-CoV-2 to determine the potential of faecal-oral transmission of the virus.

## Material and methods

### Ethical considerations and stool sample collection

This study did not involve human participants or human experimentation. The only human materials used were stool samples and throat swab samples collected from COVID-19 patients for public health purposes at the urging of the National Health Commission, People’s Republic of China.

In all, 258 stool samples were collected from laboratory-confirmed COVID-19 patients from nine provinces of China (Chongqing, Fujian, Heilongjiang, Sichuan, Shaanxi, Guizhou, Hebei, Gansuand Jilin) (Table 1), who had clinical manifestations of fever, dry cough, and fatigue. To provide more information regarding correlation of viral shedding in different tissues and disease severity, 69 throat swabs (or nasopharynx swabs) were collected in addition to stool samples from 69 COVID-19 patients from four provinces of China (20 from Shaanxi, 11 from Chongqing, 21 from Heilongjaing, and 17 from Jilin). The swabs that were found positive for SARS-CoV-2 nucleic acid using a dual target (ORF1ab gene and N gene) commercial real-time RT–PCR assay (BioGerm Medical Technology Co., Ltd, Shanghai, China) were collected. The stool samples were processed as per the following protocol. Sterilized PBS (5 mL; 0.05 mol/L, pH 7.4) was added into a 15 mL micro centrifuge tube, followed by transfer of 2 g (wet weight) of a faecal sample (about the size of broad bean) in the tube. The sample was mixed thoroughly with the PBS, and was centrifuged at 3,000 *g* for 20 min. The supernatant was used for further analysis.

**Table 1.** Epidemiological characteristics and etiological results of COVID-19 patients from nine provinces of China.

Province	Date of Onset	Age (years)	Time interval from onset to sampling (day)	Positive rate of SARS-CoV-2 nucleic acid in stool samples	Clinical classification (positive rate)				Number of cases associated with clustering outbreaks	Number of cases associated with diarrhoea
					Mild	Moderate	Severe	Critical		
Chongqing	7–31 Jan, 2020	6–63	5–25	61.5%, 8/13	3	2	3	0	unknown	unknown
Fujian	16–31 Jan, 2020	17–86	3–17	55.6%, 20/36	0	12	6	2	14	3
Heilongjiang	16 Jan–2 Feb, 2020	27–67	2–31	54.2%, 13/24	2	7	4	0	13	unknown
Sichuan	23 Jan–1 Feb, 2020	38–84	3–18	52.4%, 11/21	0	1	8	2	0	0
Shaanxi	21–29 Jan, 2020	4–66	5–13	50.0%, 10/20	2	7	1	0	3	0
Guizhou	26 Jan–6 Feb, 2020	2–80	2–14	45.0%, 9/20	0	7	0	2	6	0
Hebei	17–30 Jan, 2020	13–73	5–17	21.4%, 9/42	2	4	3	0	5	2
Gansu	8 Jan–6 Feb, 2020	1–69	4–27	18.5%, 12/65	2	4	6	0	6	0
Jilin	28 Jan, 2020	42	9	5.9%, 1/17	1	0	0	0	0	0
Total		1–84	2–31	36.0%, 93/258	12	44	31	6	47	5

### Nucleic acid detection in stool samples

Viral RNA was extracted from the supernatants of stool samples using a QIAamp Viral RNA Mini Kit (Qiagen, Valencia, CA, USA) and was stored at –80°C until further use. The stool samples were detected for SARS-CoV-2 nucleic acid using a dual target (ORF1ab gene and N gene) commercial real-time PCR assay (BioGerm Medical Technology Co., Ltd, Shanghai, China) according to the manufacturer’s instructions, which was the same method used for analysis of throat swab specimens. The positive cut-off Ct value of the kit was 38, and the negative cut-off Ct value was 40. The Ct value between 38 and 40 is called grey area, and the specimen needs to be collected again and tested again.

### Virus isolation

The stool supernatant was filtered using a 0.45 µm filter. Later, the supernatant was treated with penicillin (500 U/mL) and streptomycin (500 µg/mL). The treated supernatant was incubated at 4°C for at least 1 h. The growth medium in the cell culture tube (African green monkey kidney cell (Vero) cells that was provided by Sinovac Company) was removed, and the processed faecal suspension was inoculated into the cells along with 200 µL stool sample supernatant. The cells were incubated at 37°C and 5% CO_2_ for 1 h. After incubation, the cells were supplemented with 1.2 mL DMEM, and then incubated at 37°C and 5% CO_2_ for culture. After 7 days of incubation, cytopathic effect (CPE) was observed under inverted microscope, and the cells were harvested in case of complete CPE. If no CPE was observed after 7 days of incubation, a blind passage was performed and examination was continued for a further 7 days. Samples that showed CPE were harvested for transmission electron microscopy analysis. For microscopy, 500 µL of supernatant was inactivated with 2% paraformaldehyde; the mixture was incubated at room temperature for more than 1 h, following ultracentrifugation to enrich the virus particles which electron microscopic analysis was conducted.

### Spike gene sequencing

The complete *Spike* gene (3,822 nucleotides) of SARS-CoV-2 was amplified through long distance reverse transcriptase-polymerase chain reaction (RT–PCR) using in-house primers flanking the S gene (upstream primer: S21421F, AGGGGTACTGCTGTTATGTCT; downstream primer: S25546R, GTGCAACGCCAACAATAAGC). RT–PCR was performed using a SuperScript III One-Step RT–PCR System with Platinum Taq High Fidelity (Invitrogen, Carlsbad, CA, USA) according to the manufacturer’s instructions. The PCR products were purified using the QIAquick PCR Purification Kit (Qiagen, Hilden, Germany) and were then sequenced using ABI 3130 Genetic Analyzer (Applied Biosystems, Foster City, CA, USA).

### Bioinformatics and statistical analysis

To construct phylogenetic trees, the full-length genome of five SARS-CoV-2 samples was aligned with that of the representative beta-coronaviruses using Muscle algorithm in MEGA7 software [11]. For evolutionary analysis, we also selected the representative beta-coronaviruses in GenBank, including SARS-CoV, MERS-CoV, HKU-1, OC43, and some of the bat SARS-like coronaviruses strains, to construct the maximum-likelihood phylogenetic trees. The sequences were processed using ModelGenerator 0.85 to confirm GTR + I+G model as the best model, and the maximum-likelihood tree was constructed in MEGA7 with 1,000 bootstrap replicates. BioEdit (v7.0.9.0) was used to obtain nucleotide and amino acid sequence similarities [12]. Statistical analysis (chi-square test) was conducted using SPSS software (13.0) for each dataset.

### Nucleotide sequence accession numbers

All the eight *Spike* gene sequences obtained in this study were deposited in GenBank under the accession numbers MT263141–MT263148.

## Results

### Epidemiological characteristics of COVID-19 cases based on stool sample analysis

The ratio of male to female patients with stool samples positive for SARS-CoV-2 was 1:0.66 (56/37). The median age of SARS-CoV-2 nucleic acid positive patients was 47 years (range, 1–86 years) and that of SARS-CoV-2 nucleic acid negative patients was 41 years (range, 5–94 years). For patients whose faecal samples were positive and negative for SARS-CoV-2, the median time of intervals from disease onset to sampling was 9 days (range, 2–31 days) and 11 days (range, 1–35 days), respectively. No statistical difference was found between the two groups. SARS-CoV-2 was detected in faecal samples from all reported provinces. The provinces ranked from high to low based on positive rate were Chongqing (61.5%, 8/13), Fujian (55.6%, 20/36), Heilongjiang (54.2%, 13/24), Sichuan (52.4%, 11/21), Shaanxi (50%, 10/20), Guizhou (45%, 9/20), Hebei (21.4%, 9/42), Gansu (18.5%, 12/65), and Jilin (5.9%, 1/17) (Table 1).

### Positive rate of SARS-CoV-2 nucleic acid in stool samples

To evaluate the excretion of SARS-CoV-2 in faeces of COVID-19 patients, 258 stool samples were collected from COVID-19 patients and SARS-CoV-2-specific real-time RT–PCR was carried out. The results showed that stool samples of 93 of 258 patients were positive for SARS-CoV-2 nucleic acid, with a positive rate of 36%. Among them, the positive rate of SARS-CoV-2 nucleic acid in stool samples of critical, severe, moderate and mild cases was 54.4% (6/11), 75.6% (31/41), 26.0% (44/169), and 33.3% (12/36), respectively. Besides, the stool sample of an asymptomatic infected patient whose respiratory specimen was positive for SARS-CoV-2 was found to be SARS-CoV-2 negative.

### Relationship between SARS-CoV-2 in faeces and cluster and diarrhoea symptoms

To analyse the effect of cluster and diarrhoea on the excretion of SARS-CoV-2 in faeces, 223 of 258 cases who had cluster records and 199 of 258 cases who had diarrhoea symptoms were comparatively analysed. In the aspect of cluster, 57.3% (47/82) patients in faecal SARS-CoV-2-positive, while 46.8% (66/141) of those in faecal SARS-CoV-2-negative. Although the proportion of SARS-CoV-2-positive patients was relatively high, there was no significant difference between the two groups (*P* = 0.165). Meanwhile, in the aspect of diarrhoea, patients with faecal SARS-CoV-2-positive accounted for 7.2% (5/69), while those with SARS-CoV-2-negative accounted for 1.5% (2/130), showing no statistical difference between the two groups (*P* = 0.0503).

### Distribution characteristics of nucleic acid content in faecal specimens of COVID-19 patients

**Figure 1.**
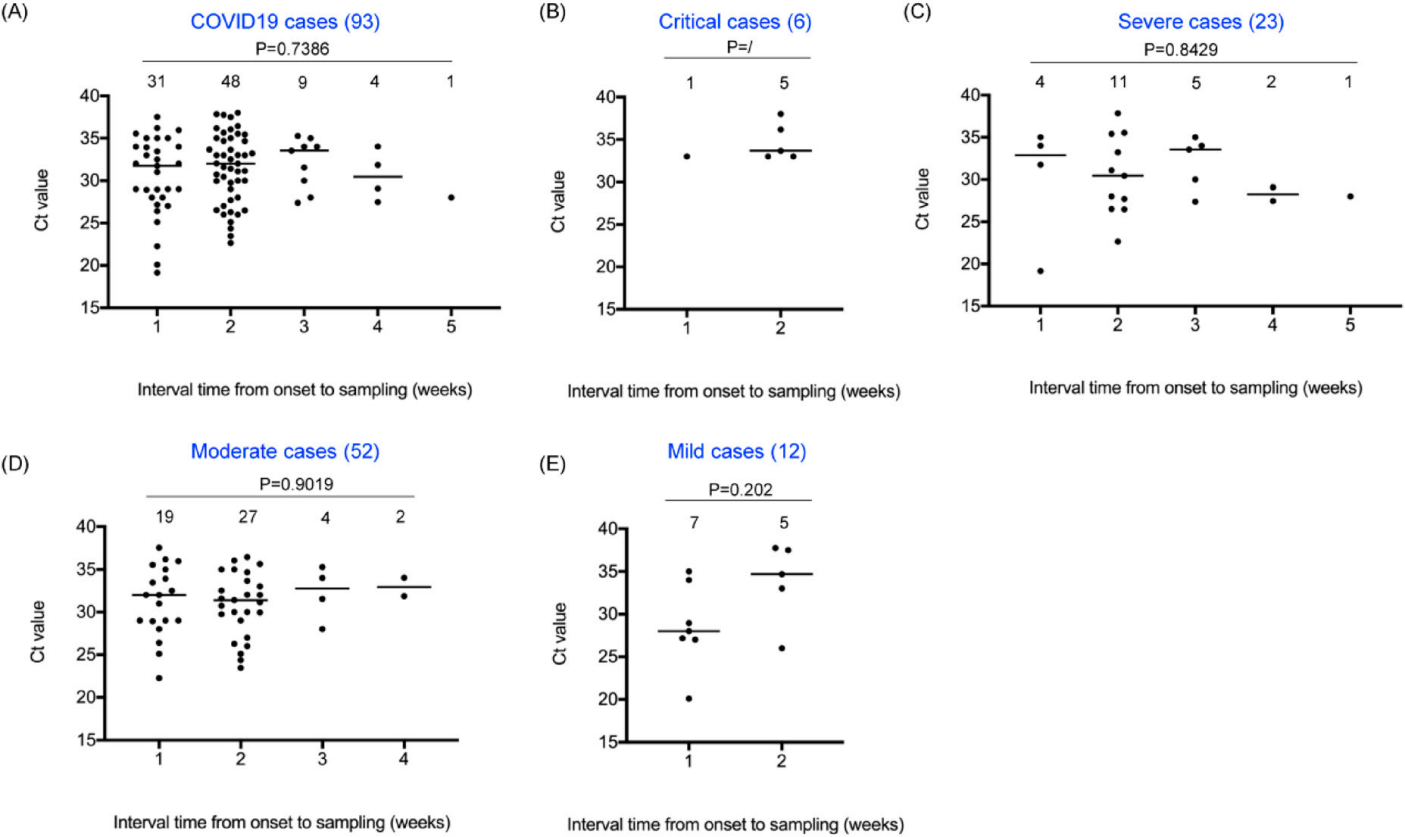
Distribution of nucleic acid content in faecal specimens of COVID-19 patients. Y-axis represents Ct values and X-axis represents the intervals from the onset to sampling (weeks). Various clinical classifications, case numbers and *P* values are shown above each of the charts. Case numbers of each interval group are indicated on the top. Ct values are marked with black solid circles. The median value of each set of data is indicated by a horizontal line.

The nucleic acid content of SARS-CoV-2 in stool specimens was widely distributed, with stool samples of 37 patients having Ct value in the range of 19–30 (37/258, 14.3%) and stool samples of 56 patients having Ct value greater than 30 (56/258, 21.7%). According to the intervals from onset to sampling, the nucleic acid of SARS-CoV-2 could be detected in the stool samples from 1 to 5 weeks after the date of onset (Figure 1). There was no significant difference in Ct value among the groups (*P* = 0.202 in mild cases, *P* = 0.901 in moderate cases, *P* = 0.8429 in severe cases, and *P* = 0.7386 in all cases), but the proportion of nucleic acid of SARS-CoV-2 detected within two weeks was higher (80.6%, 75/93), indicating a higher nucleic acid content in faeces of COVID-19 patients within two weeks after the date of onset. From the perspective of clinical classification, nucleic acid of SARS-CoV-2 can only be detected in stool samples of critical and mild patients within two weeks, while it can be detected in samples of patients with severe and moderate symptoms within a maximum of 4–5 weeks.

### Correlation between viral shedding of different samples and disease severity

To explore the relationship of viral load with disease severity in different parts of COVID-19 patients, the Ct values of throat swab and stool from COVID-19 patients of four provinces (Shaanxi, Heilongjiang, Chongqing, Jilin) were comparatively analysed. Results showed that the Ct values of throat swab in 85% (17/20) Shaanxi patients, 66.7% (14/21) Heilongjiang patients, 54.5% (6/11) Chongqing patients, and 94.1% (16/17) Jilin patients were lower than those of stool (*P* = 0.046), Ct value difference between throat swab and stool in 92.5% (49/53) cases were more than 3, indicating the viral load in the respiratory tract of these cases were significantly higher than those of feces.

**Figure 2.**
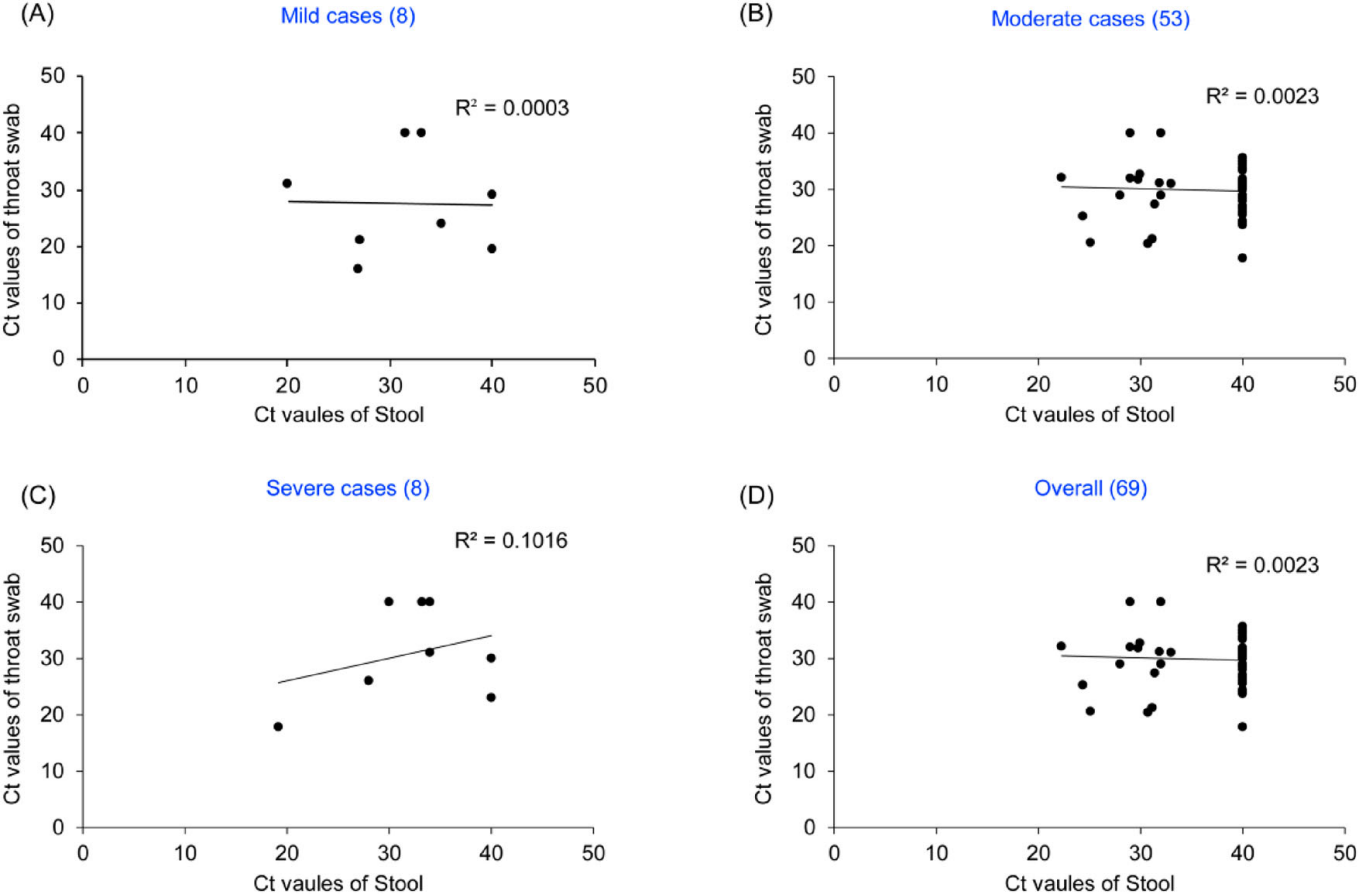
Correlation between Ct values in throat swab/stool specimens from the same COVID-19 patients and disease severity. (A) Mild cases, (B) Moderate cases, (C) Severe cases, (D) Overall. Y axis represents Ct values of throat swab and X axis represents Ct values of stool.. Various clinical classifications and case numbers are shown above each of the charts.. In this study, the CT value of the negative samples was counted as 40.

Further, We analysed the relationship of the differences of Ct value between throat swab and fecal with disease severity. The proportion of the Ct values of throat swab lower than those of stool were 62.5% (5/8) in mild cases, 81.1% (43/53) in moderate cases, 62.5% (5/8) in severe cases, indicating respiratory viral load was higher than those of fecal in different clinical classification cases. When the Ct values of all paired samples were combined according to disease severity, there was no correlation in the Ct values between throat swab and stool in any clinical classification (*R*^2^ = 0.0003 in mild, *R*^2^ = 0.0023 in moderate, *R*^2^ = 0.1016 in severe, *R*^2^ = 0.0015 in overall, Figure 2). In this study, the CT value of the negative samples was counted as 40.

### Live SARS-CoV-2 in stool samples of COVID-19 patients

**Figure 3.**
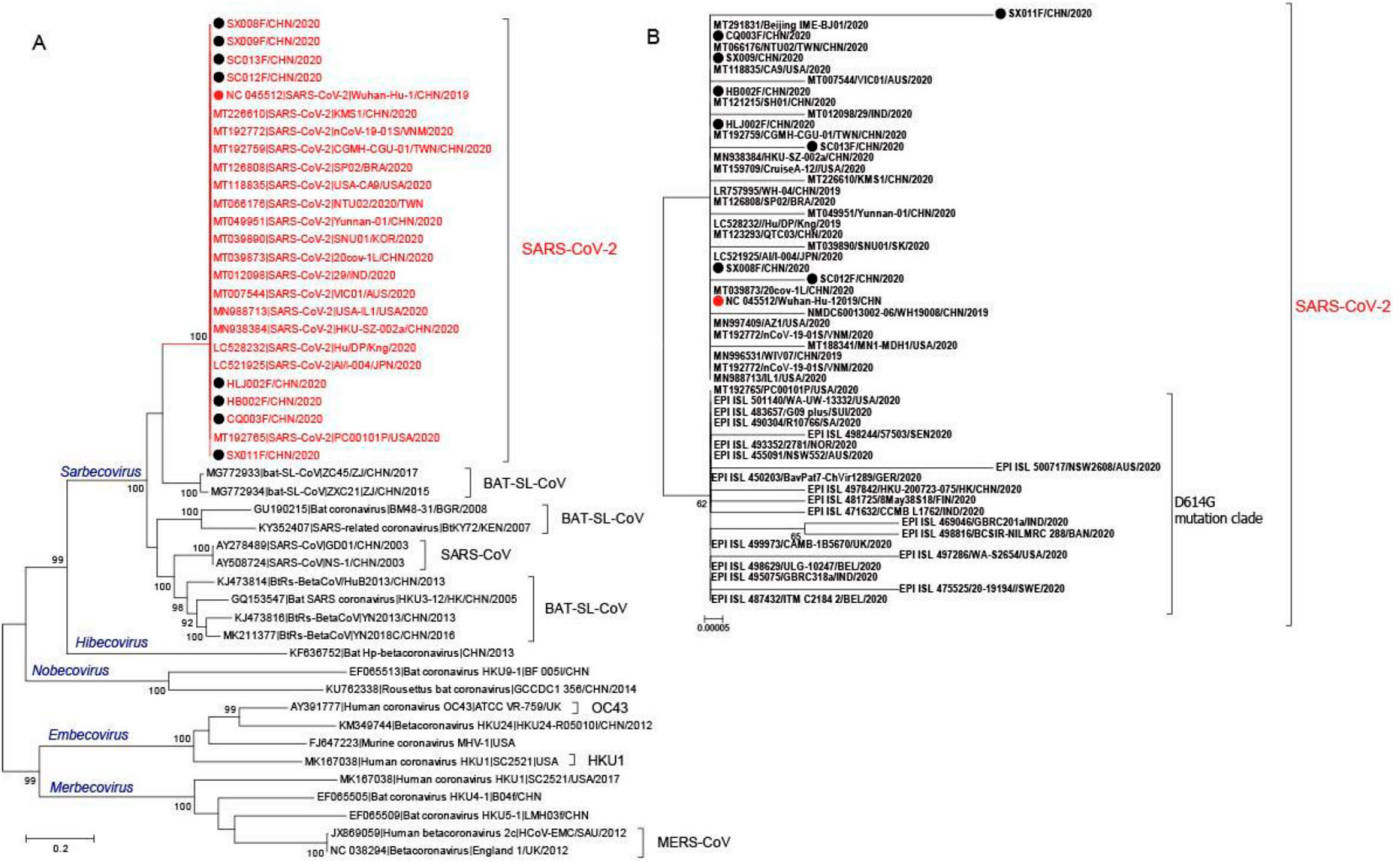
Phylogenetic analyses of *Spike* gene sequences of (A) representative SARS-CoV-2 and other beta-coronaviruses; (B) representative SARS-CoV-2 *Spike* gene sequences. The strains identified in this study are indicated by black dots, the first isolated Wuhan-Hu-1 strain (GenBank No. NC_045512) is indicated by red dot. The D614G mutation *Spike* gene sequences were further obtained from GISAID database to compare with the sequences identified in this study.

**Figure 4.**
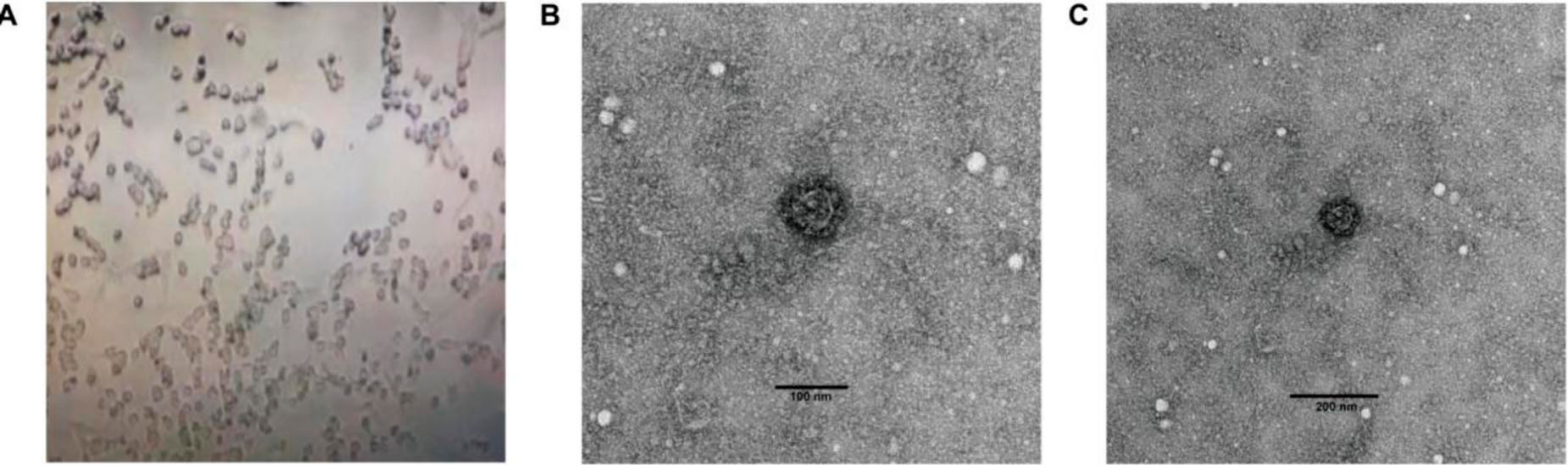
Cytopathic effect (CPE) as analysed through electron microscopy after inoculating stool suspension into Vero cells. (A) Complete CPE was observed using optical microscopy after inoculating stool suspension of strain HLJ002 into Vero cells and a virus particle with typical morphology of coronavirus was observed using transmission electron microscopy at (B) high magnification and (C) low magnification, respectively.

SARS-CoV-2 was isolated from a stool specimen (Ct value 24) of a laboratory confirmed severe COVID-19 patient (a severe case with the date of onset: 16 Jan 2020) from Heilongjiang province of China (strain HLJ002/HLJ/CHN/2020). The sequence of the full-length genome of strain HLJ002 indicated that the virus had high nucleotide similarity (99.98%) to the first SARS-CoV-2 (GenBank No. NC_045512) strain isolated from Wuhan, China (Figure 3). In the Vero cells, the virus caused obvious CPE, and the virus particles with typical morphology of coronavirus could be observed under the electron microscope (Figure 4).

### Amino acid mutations in Spike glycoprotein of SARS-CoV-2

Eight complete *Spike* gene sequences from stool specimens with low Ct value (under 24) were obtained using long distance RT–PCR. A basic analysis of the eight *Spike* gene sequences identified in this study combined with all the *Spike* gene sequences from GenBank (dated to 20 May 2020, sequences with undetermined nucleotides were eliminated) was conducted. Among a total of 338 sequences, the nucleotide and amino acid similarity varied from 98.7% to 100% and 99.0% to 100%, respectively, indicative of high genetic consistency. Only 48 polymorphic sites (two variants) were detected among all 3,822 sites, including a codon deletion of one sequence, which resulted in 31 amino acid mutations, and one deletion, suggesting a high non-synonymous mutation rate. Phylogenetic tree based on selected *Spike* gene sequences reflected high consistency in SARS-CoV-2 strains (Figure 3). Nevertheless, single base substitution of three stool samples at four nucleotide sites was detected, including a transition of C_3755_T in strain SC012F, a transversion of A_1883_T in strain SC013F, and a transition of C_149_T and transversion of C_968_A in strain SX011F. Surprisingly, these nucleotide changes all resulted in non-synonymous mutations, which were presented as amino acid mutations S_1252_F, Q_628_L, and S_50_L and T_323_K in *Spike* glycoprotein in strains SC012F, SC013F, and SX011F, respectively. Three of the amino acid mutations were found in *S1* subunit, but no changes occurred in receptor-binding domain (RBD). It is worth noting that the four nucleotide/amino acid changes detected in the three sequences in this study were not seen in other 335 sequences, which warrants the urgent need to investigate whether SARS-CoV-2 isolated from faecal samples is more likely to mutate.

It is worth noting that eight Spike sequences showed no mutation in amino acid site 614 compared with the first isolated Wuhan-Hu-1 strain (GenBank No. NC_045512), and all eight Spike sequences have a 614-D. This also proved that the early SARS-CoV-2 had no mutation at 614 (Figure 3(b)).

## Discussion

SARS-CoV-2 is a new virus with its own characteristics; the clinical characteristics of SARS-CoV-2 are different from those of SARS. Such differences, while based on limited data, may be playing a role in the apparent efficacy of rigorously applied non-pharmaceutical intervention (NPI), public health measures to interrupt chains of human-to-human transmission in a range of settings in China. SARS-CoV-2 is unique among human coronaviruses in its combination of high transmissibility, fatal outcomes in some high-risk groups, and ability to cause huge societal and economic damage. In addition, the risk of reintroduction in previously infected areas must be considered, as the animal origin of SARS-CoV-2 is not known.

SARS-CoV-2 is transmitted via droplets and fomites from close contact between an infector and infectee. Airborne spread by aerosol is possible in the presence of aerosol generating procedures in health care facilities but has not been reported for COVID-19, nor is believed to be a major driver of transmission based on available evidence. Faecal shedding has been demonstrated in some patients, and live virus has been identified in a few case reports [13]. However, the role and significance of faecal-oral transmission for COVID-19 remains to be determined.

The recognized routes of transmission of SARS are direct and indirect contact with infected individuals, close-up droplets, and aerosols generated by some diagnostic and therapeutic operations. In addition, the SARS outbreak investigation in the hospital and Taoda Garden in Hong Kong suggests that there may be a long-distance transmission of the virus aerosol contained in the toilet through the exhaust or sewage pipes under specific conditions, indicating that improper handling of the patient's stool and vomitus may result in transmission [14]. In this study, we verified the presence of SARS-CoV-2 RNA in 36% of faecal specimens in COVID-19 patients. Recent evidence suggests that hand, food, and water contamination may be caused by faecal content, and it may cause serious infection through invasion of the mouth and respiratory tract [15].

Some scholars believe that there may also be a faecal-oral route for the transmission of SARS-CoV-2 [10,16]. However, they have not yet established a complete evidence chain of infection caused by oral intake, and have not yet confirmed whether SARS-CoV-2 can cause infection through the high gastric acid barrier, and have not reported the evidence of outbreak possibly caused by polluted food or water.

We note that instances of transmission have occurred within health care settings and prisons. However, at the present time, it is not clear what role these settings and groups play in transmission. They do not appear to be major drivers of the epidemic dynamics overall. Globally, there have been reports of COVID-19 transmission in cruise ships (Diamond Princess) [17], prisons (Hubei, Shandong, and Zhejiang of China), and hospitals (as above) [18]. The close proximity and contact among people in these settings and the potential for environmental contamination are important factors, which could amplify transmission.

The ideal animal model for studying routes of virus transmission, pathogenesis, antiviral therapy, vaccine, and immune responses has yet to be found. Angiotensin converting enzyme II (ACE2) may act as the potential intermediate hosts transmitting SARS-CoV-2 to humans [19,20]. ACE2 is highly expressed not only in alveolar cells, esophageal epithelial cells, and stratified epithelial cells, but also in the absorptive enterocytes of the ileum and colon, which indicate that GI system is a potential shedding pathway of COVID-19 [8]. The ACE2 transgenic mouse model and Macaca rhesus model are already used in research laboratories. Systematic addressing of which models can accurately mimic human infection is required. If there is a suitable animal model for SARS-CoV-2, we can carry out animal experiments by inoculating SARS-CoV-2 in the stomach of the models to determine whether the virus remains alive in the intestine and to judge the oral function of faecal oral route.

In a recent study, researchers found that a D614G mutation in the SARS-CoV-2 genome enhances the virus's ability to infect human cells, which may have helped it to become the leading strain of the virus spreading around the world today [21]. Our study confirmed that there was no mutation at site 614 in the early SARS-CoV-2 strain; the mutation from 614-D to 614-G may have occurred first in Europe, then in North America and Oceania, and then in Asia, and now this mutation is shared by all viral genomes in L-lineage European branch I [22]. In view of the importance of this site, continuous surveillance is needed.

The results of this study also suggest that although live virus can be excreted from faeces and faeces can contaminate hands, environment, and articles through contact with respiratory pathway, there is no evidence that oral intake can achieve infection. However, the present method for the diagnosis of viral RNA of SARS-CoV-2 in oral swabs is not perfect because live SARS-CoV-2 may exist in faeces while oropharyngeal specimen is negative, and only negative oropharyngeal swab may not be the best indication for discharge; therefore, negative faecal viral RNA test should be added.

Determination of whether COVID-19 is transmitted through faecal-oral route (oral intake and digestive tract infection) would play an important role in the spread of disease needs further research and confirmation. However, a small number of patients have shown significant faecal detoxification capacity, and have direct or indirect contact with their hands, environment, food, water, etc. polluted by faeces. Additionally, overflow of aerosols from sewage pipes, which then invades the respiratory tract or mucous membrane, may lead to infection, and further spread of COVID-19 in families, hospitals, workplaces, public places, and communities [23].
